# Supplementary material for: Culturally adapting a mindfulness and acceptance-based intervention to support the mental health of adolescents on antiretroviral therapy in Uganda
Source: PLOS Glob Public Health. 2023 Mar 7;3(3):e0001605. doi: 10.1371/journal.pgph.0001605 (PMC10021405; doi:10.1371/journal.pgph.0001605)
Supplement: S1 Text — (DOCX) [file pgph.0001605.s001.docx]

**ACT for Adolescents Study**

Interview guide for Users (Adolescents)

This interview will last between 20 to 30 minutes.

**Qn1;** How do you feel after going through the training sessions? Why? Is there anything that caught your attention most or confused you? What could that be? And how?

**Qn2**; How easy or hard was it for you to understand things we have looked at? Why?

**Qn3**; What might you miss out on by trying to use what you have learnt from these training sessions? Why?

**Qn4**; Do you think what we have covered in the training is useful to you? how? What immediate benefits have you received?

**Qn5**; How do you think this training works?

**Qn6;** How confident are you that you will keep practicing what you have learnt when dealing with difficult thoughts or experiences? Why?

**Qn7**; Do you find this training to be appropriate for people of your age? Why? Would you recommend others to take on the training? Why?

Thank you for participating
