# Supplementary material for: Culturally adapting a mindfulness and acceptance-based intervention to support the mental health of adolescents on antiretroviral therapy in Uganda
Source: PLOS Glob Public Health. 2023 Mar 7;3(3):e0001605. doi: 10.1371/journal.pgph.0001605 (PMC10021405; doi:10.1371/journal.pgph.0001605)
Supplement: S2 Text — (DOCX) [file pgph.0001605.s002.docx]

COREQ (Consolidated criteria for Reporting Qualitative research) Checklist

A checklist of items that should be included in reports of qualitative research. You must report the page number in your manuscript where you consider each of the items listed in this checklist. If you have not included this information, either revise your manuscript accordingly before submitting or note N/A.

| **Topic** | **Item** **No.** | **Guide** **Questions/Description** | **Reported** **on**  **Page** **No.** |
| --- | --- | --- | --- |
| **Domain** **1:** **Research** **team**  **and reflexivity** | | | |
| *Personal* *characteristics* | | | |
| Interviewer/facilitator | 1 | Which author/s conducted the interview or focus group? **Three members of the research team that did not participate in training participants (they are not listed as part of the co-authors)** | Pg. 14 |
| Credentials | 2 | What were the researcher’s credentials? **Data collectors were trained in mindfulness and acceptance-based therapies and were Psychology graduate students.** | Pg. 14 |
| Occupation | 3 | What was their occupation at the time of the study? **They were fulltime students not engaged in any other form of work**. | Pg. 14 |
| Gender | 4 | Was the researcher male or female? **Both** **3 males and 1 female** | Pg. 14 |
| Experience and training | 5 | What experience or training did the researcher have? **Data collectors were** **trained in mindfulness and acceptance-based therapies** | Pg. 14 |
| *Relationship* *with*  *Participants* | | | |
| Relationship established | 6 | Was a relationship established prior to study commencement? **Yes it was established at recruitment** | Pg. 14 |
| Participant knowledge of  the interviewer | 7 | What did the participants know about the researcher? e.g. personal  goals, reasons for doing the research? **Researchers made self-introductions** |  |
|  |  |  | Pg. 14 |
|  |  |  |  |
| Interviewer characteristics | 8 | What characteristics were reported about the inter viewer/facilitator?  e.g. Bias, assumptions, reasons and interests in the research topic? **Social desirability bias which arise when trainers conduct the interviews** |  |
|  |  |  | Pg. 15 |
| **Domain** **2:** **Study** **design** | | | |
| *Theoretical* *framework* | | | |
| Methodological orientation and Theory | 9 | **What methodological orientation was stated to underpin the study? e.g. grounded theory, discourse analysis, ethnography, phenomenology,**  **content analysis**. **A qualitative methodological framework using thematic analysis** |  |
|  |  |  | Pg.15 |
| *Participant* *selection* | | | |
| Sampling | 10 | How were participants selected? e.g. purposive, convenience,  consecutive, snowball? **Convenience sampling** |  |
|  |  |  | Pg. 13 |
|  |  |  |  |
| Method of approach | 11 | How were participants approached? e.g. face-to-face, telephone, mail,  Email? **Face to face by their health-care providers** |  |
|  |  |  | Pg. 13 |
|  |  |  |  |
| Sample size | 12 | How many participants were in the study? **Nine (9) participants** | Pg. 14 |
| Non-participation | 13 | How many people refused to participate or dropped out? Reasons? **None** | Pg. 13-14 |
| *Setting* | | | |
| Setting of data collection | 14 | Where was the data collected? e.g. home, clinic, workplace**: Clinic** | Pg. 13 |
| Presence of non-  Participants | 15 | Was anyone else present besides the participants and researchers? **No. It was an individual in-depth interview** |  |
|  |  |  | Pg. 14 |
|  |  |  |  |
| Description of sample | 16 | What are the important characteristics of the sample? e.g. demographic  data, date: **Adolescents 15-19-year-old adolescents living with HIV/AIDS** |  |
|  |  |  | Pg. 13-14 |
|  |  |  |  |
| *Data* *collection* | | | |
| Interview guide | 17 | Were questions, prompts, guides provided by the authors? Was it pilot  tested? **Yes, an interview schedule that was pilot tested was used.** | Pg. 13 |
|  |  |  |  |
| Repeat interviews | 18 | Were repeat interviews carried out? If yes, how many? **No repeat interviews were conducted.** | N/A |
| Audio/visual recording | 19 | Did the research use audio or visual recording to collect the data? **Audio recordings**. | Pg. 14 |
| Field notes | 20 | Were field notes made during and/or after the interview or focus group? **Yes, they were made during and after interviews** | Pg. 15 |
| Duration | 21 | What was the duration of the interviews or focus group? **25 - 30 minutes** | Pg. 14 |
| Data saturation | 22 | Was data saturation discussed? **A guide on “how many interviews are enough to reach saturation” was followed.** | Pg. 14 |
| Transcripts returned | 23 | Were transcripts returned to participants for comment and/or |  |

| **Topic** | **Item** **No.** | **Guide** **Questions/Description** | **Reported** **on**  **Page** **No.** |
| --- | --- | --- | --- |
|  |  | correction? **No** | N/A |
| **Domain** **3:** **analysis** **and**  **Findings** | | | |
| *Data* *analysis* | | | |
| Number of data coders | 24 | How many data coders coded the data?  **Two** | Pg. 15 |
| Description of the coding  Tree | 25 | Did authors provide a description of the coding tree? **Yes** |  |
|  |  |  | Pg. 15 |
| Derivation of themes | 26 | Were themes identified in advance or derived from the data? **a** ***Priori* themes”** | Pg. 15 |
| Software | 27 | What software, if applicable, was used to manage the data? **NVivo (version 12)** | Pg. 15 |
| Participant checking | 28 | Did participants provide feedback on the findings? **No** | N/A |
| *Reporting* | | | |
| Quotations presented | 29 | Were participant quotations presented to illustrate the themes/findings?  Was each quotation identified? e.g. participant number. **Yes** |  |
|  |  |  | Pg. 21-24 |
|  |  |  |  |
| Data and findings consistent | 30 | Was there consistency between the data presented and the findings? **Yes** | Pg. 21-24 |
| Clarity of major themes | 31 | Were major themes clearly presented in the findings? **Yes** | Pg. 21-24 |
| Clarity of minor themes | 32 | Is there a description of diverse cases or discussion of minor themes? **No** | N/A |

Developed from: Tong A, Sainsbury P, Craig J. Consolidated criteria for reporting qualitative research (COREQ): a 32-item checklist for interviews and focus groups. *International* *Journal* *for* *Quality* *in* *Health* *Care*. 2007. Volume 19, Number 6: pp. 349 – 357
