# Supplementary material for: Culturally adapting a mindfulness and acceptance-based intervention to support the mental health of adolescents on antiretroviral therapy in Uganda
Source: PLOS Glob Public Health. 2023 Mar 7;3(3):e0001605. doi: 10.1371/journal.pgph.0001605 (PMC10021405; doi:10.1371/journal.pgph.0001605)
Supplement: S1 Data — (DOCX) [file pgph.0001605.s003.docx]

ACT for Adolescents Project

Intervention Adaption Recording Form

Group: 1

Total number of participants: 5

| What is modified? (Including page number) | Suggested adaptation | Reason for adaptation being made | Bernal category of adaptation | How decision is reached |
| --- | --- | --- | --- | --- |
| Videos in all sessions | Should be replaced with skits led by facilitators to replicate what is in the videos. They don’t have to be similar to the video exactly i.e same content but should communicate the main concept. | - It will make it more participatory - For when the videos aren’t available due to common issues in Uganda like power cuts, no generators, etc - To cover language issues | Methods | Voted 5/5 |
| Session 1. Pg 8  “Using Social DNA-v to form the group” | Facilitators should be trained on values work; how to recognize the values because the participants randomly say sentences and the facilitator has to reframe them as values. | Not to mistake morals for values | Methods | 4/5 |
|  | Overall training to understand the nuances, what should be picked from the adolescents as they might not know how to discuss their feelings. | For example, facilitators might not have an understanding on how to identify, “what is the noticer? what is in the noticer” | Methods | 5/5 |
|  | Besides value and strengths cards, emotional cards should also be made (with faces e.g sad faces). | - To help participants and facilitators both to put a name to their emotions - Facilitators should have a guide to know emotions described by adolescent. - Much as emotions are universal, one may not know what they are feeling is called but can point it out. | Methods  Concepts | 5/5 |
| All items presented to adolescents e.g cards, roleplays, games | - Translate all items presented directly to adolescents. If not on paper, then facilitators should be able to switch the language. - Simplify/break down the language – for example some items on the strength cards were a bit complicated. | - For easy understanding by adolescents. - Some cards had complicated language. | Language | 5/5 |
| Session 1: “Introducing yourself” – Pg 7 | - Facilitators’ introductions should be dependent on contextualized rapport. - Train facilitators on how to say the introductions i.e should be friendly. - Ensure adolescent friendly facilitators in their dress code, language and demeanor. | - Too cold - We work well with people we have a connection with especially for adolescents. - To build relatability. | Methods & Concepts |  |
|  | Can a session be broken into 2, weekly? | Not to leave anyone behind. | Methods | 3/5 |
|  | Recaps should determine whether facilitator should continue to next session. And ask adolescents if session can be repeated. |  | Methods | 1/5 |
|  | Sessions should be paced. | - Some adolescents come to the clinics once in 6 months others might not come at all. - 6 sessions, 2 hours each might not be possible based on how appointments are done at the health facilities. | Methods & Context | 4/5 |
|  | Talk to the clinics’ heads and inform them that the program will be implemented weekly. | To ensure adolescents turn up weekly not based on appointments. | Methods | 4/5 |
|  | Consider school curriculum and logistical part i.e transport. | Because logistics affect adherence. | Context | 5/5 |
|  | Maintain 6 sessions but;   - Prepare adolescents from the beginning i.e we shall be having 6 sessions and each will run for 2 hours, and this will be done in the sessions. This wasn’t mentioned anywhere yet very key. - From the beginning of every session, the facilitator should inform adolescents the present session number and how many are remaining e.g … two more sessions to go. | To prepare young people mentally. | Methods & Context | 5/5 |
| Session 6 – Pg46 | The last session should not only be about evaluation but also preparation i.e   - What have you learnt and how will you continue practicing? | - To avoid relapse. - Maintain the work/what has been gained. - For continuity. | Methods | 3/5 |
| Home tasks | - Facilitator should engage adolescents when doing the homework i.e - Find out if it suits their goals. - Is it useful and doable? - Ask if the HW is something they’ll be able to do and if it makes sense. - Summarise and keep it simple e.g in session one, “this week when you’ve a choice to make I would like you to notice how often it happens, if it’s difficult or easy…” - Tailor the homework to their experience i.e adherence behavior if possible. - Ask for feedback; what do you think of what’ve discussed? | To ensure that it is done but not to please the facilitators but with understanding on how it helps them.  Because some exercises like breathing in and out are new in our culture.  It’s a mouthful.  Because of difference in culture. Children in the western world are already conversant with most of the things in the protocol but not Ugandans. | Methods & Concepts | 4/5 |
| The seaweed metaphor, Pg 16-17 | - Use local examples. - Localise the expressions and metaphors. | Easy relatability and understanding. For example, most adolescents haven’t even been to the lake. | Metaphors | 5/5 |
|  | There should be room for disclosure by facilitators, put it in your own life experiences.   - Examples of where you’ve applied it and if it worked as a facilitator. - Share some of your experiences e.g “when I tried mindful breathing it was funny at first but later…” - Give relatable examples. | So, the adolescent can relate with you and the program, go the process with them. | Methods | 4/5 |
|  | Facilitators MUST be trained on the developmental phases e.g adolescence and show them that they can relate. |  | Methods & Concepts | 3/5 |

Other Comments

- If sessions are compressed and delivered at a shorter time, then it will be more educational than therapeutic. We need to be able to deliver but also create change.
