# Supplementary material for: Culturally adapting a mindfulness and acceptance-based intervention to support the mental health of adolescents on antiretroviral therapy in Uganda
Source: PLOS Glob Public Health. 2023 Mar 7;3(3):e0001605. doi: 10.1371/journal.pgph.0001605 (PMC10021405; doi:10.1371/journal.pgph.0001605)
Supplement: S2 Data — (DOCX) [file pgph.0001605.s004.docx]

ACT for Adolescents Project

Stakeholders’ workshop

Group: 2

It was observed and suggested that:

1. The number of members in a group should be between 6 -12 rather than 10-20. This helps to decongest a group.
2. Since the age range of the group members is 15 – 19 years, only those adolescents aged 15 -17 years should be in separate group from those who are aged 18-19 years. The reasons fronted for such grouping included that the first category are minors and can assent while the second is made up of young adults who can consent; they maybe in different classes and therefore with different developmental experiences.
3. It may be okay to mix girls and boys because both sexes share the HIV status. Also, it might bring about one sex learning from the other in terms of their (unique) experiences.
4. It would be better to train counselors at the sites of intervention before they can deliver the psychotherapy. This would be an opportunity for capacity training. However, another member proposed pairing site counselor/s with graduate psychosocial trainee/s to ensure that the intervention is implemented with fidelity.
5. Rationale for all suggested modifications: To make the ACT-DNA-V understandable and relatable; to achieve intervention goals; and to attain cultural as well as age sensitivity and appropriateness.
