# Supplementary material for: Culturally adapting a mindfulness and acceptance-based intervention to support the mental health of adolescents on antiretroviral therapy in Uganda
Source: PLOS Glob Public Health. 2023 Mar 7;3(3):e0001605. doi: 10.1371/journal.pgph.0001605 (PMC10021405; doi:10.1371/journal.pgph.0001605)
Supplement: S3 Data — (DOCX) [file pgph.0001605.s005.docx]

ACT for Adolescents Project

Intervention Adaption Recording Form

Group: 2

Total number of participants: 5

| What is modified? (Including page number) | Suggested adaptation | Reason for adaptation being made | Bernal category of adaptation | How decision is reached |
| --- | --- | --- | --- | --- |
| The language in which the entire protocol is being presented (English) | - The entire protocol needs to be translated to a language which resonates with the targeted adolescents, localize the protocol | - Some adolescents are not educated enough while for other young people these are not day to day words to the adolescent. | language | 5/5 |
| Seaweed | - Kitoogo, ekiddo and swamp weed | - Before putting the protocol out, engage the young people for common words they easily understand and relate with. | language | 4/5 |
| The game of life  Page 9 | - Embezzle money can be replaced with steal money - Scrooge can be replaced with be mean - Get fired replaced with get chased from work. - Be a hermit replaced with be a loner | - Most Ugandan adolescent are not Conversant with most of the things in the protocol. | language | 4/5 |
| Survivor game  Page 21 | Imagine you want to take a walk past curfew time and the fear of facing the police  Running away from school and the trouble that comes with making the decision. | - The warrior metaphor is a little farfetched, they need to be Ugandan based | metaphor  metaphor | 4/5 |
| The free hug  Page 16 | The free hug can be replaced with a free sign for peace kubonga.  In the free hug the characters should be black | It’s not cultural acceptable or common females and males to hug out of the blue  Black characters will help the adolescent to relate and be open about there emotions. | metaphor | 5/5 |
| The Noticer game  Page 18 | Don’t change but have them in brackets  Emoter (feeler)  Noticer (Obsever) | The word may not make meaning to the adolescent | metaphor | 4/5 |
| values | - Tailor some of the examples to HIV and ARV adherence. | - It’s important to contextualize the examples so that therapy goals are in line with the adolescent goals. | goals | 5/5 |
| Six ways to wellbeing  Page 49 | - Address structural constraints around the adolescent. - Like the family and how it affects the adolescent. | - The well being ways are too westernized | Context | 5/5 |
| Poem for magic adviser sounds like activity  Magic  Page 25 | - Incorporate local poems with local characters history. E.g. nambi and kintu | - These are characters adolescents can relate with, they have mental representations of these characters from child hood | metaphor | 5/5 |
| Items like the value cards.  Page  Page 43 | - Being kind(show a picture of an adolescent helping the homeless or helping blind or the elderly cross the road ) - Cards Should be translated directly to the local language by the facilitator | - The card should be tailored to things that adolescent in the appropriate region can easily relate to | Language \|Methods | 4/5 |
|  | Provide alternative protocol for illiterate adolescents. | - Some may not be in position to write or may be even disabled | language | 2/5 |
| Home work | - Explain choices in the context of given boundaries - Help adolescents internalize and encourage them to apply what really works for them. | - Adolescents are still under the roof of their parents, they don’t have much of a choice in some areas of their life yet, choice may apply in one home but never apply in another home | Methods / Context | 3/5 |
