# Supplementary material for: Culturally adapting a mindfulness and acceptance-based intervention to support the mental health of adolescents on antiretroviral therapy in Uganda
Source: PLOS Glob Public Health. 2023 Mar 7;3(3):e0001605. doi: 10.1371/journal.pgph.0001605 (PMC10021405; doi:10.1371/journal.pgph.0001605)
Supplement: S4 Data — (DOCX) [file pgph.0001605.s006.docx]

**NOTES FROM MEETING WITH ADOLESCENTS LIVING WITH HIV**

**Group discussion rules as given by the adolescents**

1. Put phones in silent mode
2. Non judgemental
3. Respect others’ ideas
4. Active participation
5. Engagement
6. Team work
7. Be audible enough
8. Don’t laugh at others’ responses
9. Confidentiality

**Observations**

- Values were not identified easily by the adolescents, they need help. Most were confusing values with hobbies or likes.
- Adolescents loved story telling – should be considered under time for the sessions.
- Some adolescents preferred to speak out their values instead of writing them.
- They were finding difficulty reconciling their values and what has been done or needs to be done in service of those values. Gratitude is an important value and in service of that value he sent out emails…
- ACCEPTING MYSELF – was a value that cut across as they related this to their status.
- Religion/God all the adolescents mentioned as important in (their) life.

**Feedback from the adolescents**

- It’s a helpful programme but adapting it to live a better life will depend on the individual.
- Using it at school is not fair. People will say those with HIV have gathered in their group. We should leave it at the clinic.
- Fellow adolescents should also participate in instructing in this program. When people like you in ties keep talking to us we may not open up well because we think you don’t understand our problems.
- Some of these activities can be done in our drama clubs especially on adolescent clinic days.
- “For the different advisors, can’t the silent and obedient advisor save one from danger or they are just useless?”
- Not every item on the cards made sense.

**Suggestions on the Game of Life**

What is missing?

1. Interaction i.e talking with others
2. Connection (with others)
3. God fearing
4. Unite the world (unity)
5. Family
6. Music (Listening/making/instruments)

What is confusing/not understood and what it should be replaced with

| 59 – being grumpy (being short tempered) | 27 - Gamble |
| --- | --- |
| 54 – You stink | 20 – Drop out |
| 38 – face ruin (lose everything) | 7 – graduate high school (complete secondary school) |
| 15 – Angry | 43 – Become a scrooge |
| 42 – fight for peace | 23 - Persist |
| *2 – Get deported | 16 & 53 – after explaining they understood |
| 48 – be a hermit (be alone) | 4 – Cheat others |
| 50 – Lie compulsively | *22 – Be impulsive |

*to be removed

Other Comments

Adopt ndaga buttoned for biological DNA and ndaga yeneyisa for DNA-v
