# Supplementary material for: Culturally adapting a mindfulness and acceptance-based intervention to support the mental health of adolescents on antiretroviral therapy in Uganda
Source: PLOS Glob Public Health. 2023 Mar 7;3(3):e0001605. doi: 10.1371/journal.pgph.0001605 (PMC10021405; doi:10.1371/journal.pgph.0001605)
Supplement: S5 Data — (DOCX) [file pgph.0001605.s007.docx]

**Rules**

- Put all phones in silence.
- There should active participation
- Everyone must be engaged
- There should be team work
- Non judgement
- Be loud
- Observe confidentiality

**Feedback on the game of life**

Missing (thing that should be added)

- Connecting with others
- Unite the world
- Have a family
- Playing instruments.

**Replacements**

- 48(Alone)
- 50(Alone)
- 59(short tempered)
- 43(Stingy)
- 38(lose everything)
- 23(Never give up)
- 22(dropped)
- 21(like others)

**Notes**

- Values don’t come out well, adolescents need support to identify values
- Most values written on cards don’t resonate well or create clarity we might have to develop new values
- Accepting is a common value
- The warrior metaphor was well understood and it made a lot of sense to th adolescents
- The push paper was received well and the teen were able the related well with it.
- The noticer and advisor was easily understood compared to the values
- The only would be limitation to the program is the adolescents ability to concentrate though out the session.
- Explain the advisor well so as not to confuse the meaning.
- This cannot be used in schools. People will say whoever goes to that group has HIV. They will get to know us and start using that information to make us feel bad. Its better we receive it from the clinic.
