# Supplementary material for: Culturally adapting a mindfulness and acceptance-based intervention to support the mental health of adolescents on antiretroviral therapy in Uganda
Source: PLOS Glob Public Health. 2023 Mar 7;3(3):e0001605. doi: 10.1371/journal.pgph.0001605 (PMC10021405; doi:10.1371/journal.pgph.0001605)
Supplement: S6 Data — (DOCX) [file pgph.0001605.s008.docx]

| **ACT for Adolescents Project**  **Intervention Adaption Recording Form**  **Group:** Three (3)  **Number of Participants:** Five (5) |
| --- |

| **What is modified? (Including page number)** | **Suggested adaptation** | **Reason for adaptation being made** | **Bernal category of adaptation** | **How decision is reached** |
| --- | --- | --- | --- | --- |
| The classification of the study group. | Its paramount to clarify whether the research is targeting school going children or drop outs. | Its enables participants to cognitively with others.  It also helps to properly process the language used especially in the group therapy since it’s a major requirement. | Context | 4/5 |
| There is a Need to have a Pre selection Tool | Categorization of the target audience | It will help to identify clearly the group of adolescents the research is targeting. | Methods | 4/5 |
| The game of life – Its complicated, need to be adapted to the language and symbols that can be well understood by the target group. | The team should think about something clear and simple for the adolescents to comprehend. | It is basically important to allow cognitive and experiential processing for the groups | Methods | 3/5 |
| The Board Video - The English is complicated and may not be understood (Being Yourself)  The Video of Free Hugs need to be thought through. | Create boxes or any other symbols in order to make it culturally appropriate    Create something experiential like card boards  The language also needs to be simplified.  Think about something that is convenient for our society. May be shaking hands etc. | Better understanding and processing of the concepts by the adolescents.  The cultural boundaries in Africa May limit the free hugs video. Its culturally sensitive.  Also given the pandemic it may not be appropriate. | Metaphor and Language  Metaphor | 5/5  4/5 |
| The time frame for the sessions | The two (2) hours apportioned for each session is to much for adolescents, the recommended time is usually 1 hour and 30 minutes per session or below. | The two hours are very cognitively tasking in case it is carried on, there is need to consider incorporating more experiential activities. | Methods | 3/5 |
| Home Tasks | Its paramount to think about what works for adolescents, it should be user friendly, developmental and appropriate | To enable them accomplish the tasks on time and do it wholeheartedly. | Methods | 4/5 |
| The question on phones, page 14. Needs to be revised. | Do all adolescents have phones? Can they handle the phone assignments, may be another method description instead of phones? | Adolescents have their inner drives and are curious, every time they want to find out what happens on their phones. | Methods | 3/5 |
| The Discussion on the cognitive expression of feelings, page 15. | Let us use illustrations to get their feelings step by step. | The adolescents may struggle to understand it, think about something easy for them to understand. | Language | 4/5 |
| The metaphor on page 17. | Let’s consider putting something children are familiar with in Ugandan context, have illustrations when explaining to adolescents. | The pupils may not be well versed with the pools talked about in the manual. | Metaphor | 4/5 |
| The abstract of mindfulness on page 18. | Give guiding principles through illustrations so that they can reflect on them  Be very practical in which ever step you are considering to take. | The whole process is cognitive, it may be very difficult for adolescents to process. | Language | 5/5 |
| The metaphor on page 18 is abstract. | It should be developmentally appropriate | To avoid boredom and easy processing by the adolescents. | Metaphor | 4/5 |
| The Worrier game on page 21 | It requires role plays and be integrated among adolescents.  It needs step by step processing.  Use illustrations and activities that would help them in processing. | Its important to get the concepts clearly | Methods | 3/5 |
| Pen and WOO on page 23 | It requires to use something appropriate | It doesn’t make sense at all and it’s not related | Methods | 3/5 |
| The strength cards on page 34 | The version needs to be simplified; the message should be easily communicated | It will help the adolescents get it properly and understand it | Methods | 4/5 |
| The Value cards | The messages written on the cards should be culturally appropriate, better understood and communicated. | It will allow simplicity of the process flow once well understood by the target group. | methods | 3/5 |
| **Discussion notes from DNA-V manual Review:**   1. The issue of specificity in regards to the age groups need to be thought through for consideration since they all have different development curve and the reasoning may differ especially when it comes to comprehension. 2. The issue of informed consent especially where minors are involved in the target groups 3. The issues related to gender (The question of separating boys from girls) 4. In case we are to conduct group therapy the groups should be between 10 – 15 pupils 5. The issue of training the implementors, should it be service providers at the facilities or counselors out sourced elsewhere? What about the qualifications 6. The site counselor being paired with the graduate trained counselor for support and better skills set 7. The idea of utilizing the adolescent clinics at the health facilities with the services providers – In case they need extra training it should be accorded them for better implementation.   **Challenges:**   1. There was limited time portioned for the group discussions. 2. The participants were agreeable to the opinions of one dominant expert. | | | | |
