# Supplementary material for: Culturally adapting a mindfulness and acceptance-based intervention to support the mental health of adolescents on antiretroviral therapy in Uganda
Source: PLOS Glob Public Health. 2023 Mar 7;3(3):e0001605. doi: 10.1371/journal.pgph.0001605 (PMC10021405; doi:10.1371/journal.pgph.0001605)
Supplement: S7 Data — (DOCX) [file pgph.0001605.s009.docx]

**Workshop recording**

Key highlights:

- Area we are modifying
- Suggested adapted area
- Reason for modification

SESSION 1

- Video 1 (Being yourself): needs to be contextualized or find another one which is culturally appropriate. Justification: The English in the video might be hard to be understood by adolescents. Important to use a video with an African touch which resonates with adolescents in Kampala. It could even be acted out with boxes or cardboards to create direct meaning.
- Language; the entire protocol be simplified to basic English at primary school level so that it is easily understood, Justification; compression and understanding need to be aided.
- Video 2 (free hugs): Contextualize it and also simplify the language: some posters are written in Spanish which is basically not used in Uganda.
- Clearly state the target population e.g. school going adolescents or even adolescents at home. The current state of the protocol cannot be understood by uneducated adolescents.
- Exclusion criteria: Clearly clarify the category of adolescents who are going to be in the respective groups. Is it adolescents with HIV or those with mental disorders? Adolescents with other underlying mental health challenges should be excluded, also those with mental and developmental delays should be excluded.
- The number of adolescents in each group should be between 6-12, not more than that.
- Adolescents included in session should be assessed for underlying mental health conditions before inclusion in study.
- The game of life/choice: the game of life/choice: The acting should be more than talking (p8). Its delivery should be adapted to help adolescents process its meaning in the quickest way possible. Choices identified in game of life should be closer to things adolescents are familiar with e.g. no. 48, 50 and 43. Choices and language should also be age appropriate.
- Sessions should have more visual aids and plays than presentations. The should also nor exceed 1 and 30 minutes. If its stretched to two, then activities need to be more experiential.
- Page 11, use more visuals and illustrators and make it developmentally appropriate.
- Home tasks: make them more adolescent friendly so that they can participate or accomplish them willingly.
- Page 14: Illustration of a phone, do they have phones? Can adolescents easily relate with phones.
- Page 15: the cognitive expression will not work for adolescents here. There is need to use illustrators relating to feelings because children struggle with cognitive illustration of feelings. Think about how adolescents in Uganda bring out their feelings.
- Page 16: Free hugs: look for something which will not confuse students. Cultural boundaries on certain aspects need to be considered.
- Page 17: See weed plant is unfamiliar territory; use metaphors known to adolescents in Uganda. Metaphors like swamps or running rain water seem closer.
- Page 18: step by step slowed process of teaching mindfulness in important. This is not something we are very familiar with: exercises like body scan and other mindfulness exercises be redesigned to be culturalized.
- Processing, illustrations and experiential exercises be intensified in the entire process of mindfulness if it’s to be delivered in two hours.
- Home tasks be accompanied with illustrations to aid practice.
- Page 20: the metaphor is too abstract: clarity on how to normalize and also making it developmentally appropriate.
- Page 21: warrior game: It should be acted out in a simplistic way: in explaining the different advisors, identify examples which are familiar.
- Relate “Pen says woo” to Ugandan context
- Page 34: Strength cards: the language needs to relate to things that are familiar to a Uganda adolescent. Simplify the language / words.
- Page 35: “Try it” “Track it” needs illustrations and local examples
- Page 40: value cards: the messages need to be culturally appropriate.

General comments

- Translate the videos to a local language most understood
- Make games and metaphors culturally appropriate
- Bring the HIV context e.g “can someone swallow this pill for me”
- Be mindful of the developmental stages
- Categorize adolescents according to their age groups and each group have between 6-12 members. Create a 15-17 group, and 18-19 group.
- Its fine to mix the gender but proper justification for mixing is important.
- Train a team of therapist who should deliver the sessions
- Mobilize on-site counselors and pair them with therapists during sessions.
