# Supplementary material for: Culturally adapting a mindfulness and acceptance-based intervention to support the mental health of adolescents on antiretroviral therapy in Uganda
Source: PLOS Glob Public Health. 2023 Mar 7;3(3):e0001605. doi: 10.1371/journal.pgph.0001605 (PMC10021405; doi:10.1371/journal.pgph.0001605)
Supplement: S8 Data — (DOCX) [file pgph.0001605.s010.docx]

**STAKEHOLDERS REVIEW MEETING- Adolescents living with HIV.**

Seven (7) adolescents who participated at generation also did the review.

Aim of the meeting was to cross check with adolescents if the modifications that had been done to the DNA-V manual were a representation of their ideas they suggested during generation. It was also aimed at identifying if the manual was in a better useable form to them. We also created room for more suggestions to guide re-modification.

Process: Participating where taking through the modified manual, highlighting what has been changed and the reason why it was changed. After presentation from the research team, participants formed two groups (one of four people and the other of three people) and began to discuss the modification and making more suggestions. We agreed from the onset that a group should be able to come up with consensus on the final changes to be made. In the event that consensus was had to get, they would go with majority decision. Below are the suggestions.

| Category | Modified item | What is suggested | Rationale |
| --- | --- | --- | --- |
| Values | Seeking pleasure | Find a better picture that brings meaning | Eating food does not necessary represent pleasure. We do it because we are hungry. |
|  | Being truthful | Find an alternative picture | The picture is more of irony, not clear |
|  | Asking for help | A typical black person asking for help is a better alternative | Other than the paper placed on the head, aspect of help does not come out. Picture is not a match. |
|  | Caring for myself | Replace the picture say with someone bathing | Listening to music does not bring out care aspect well. |
|  | Accepting myself | An alternative picture. Possibly someone with disability but acting useful | Photo demonstrates something else |
|  | Seeing possibilities | An alternative | Aspects of possibilities not well represented |
|  | Independent | An alternative | Picture does not match the narrative |
|  | Love | Possibly young people embracing one another | Old people are used. |
|  | Working hard | An alternative | Pic shows child labor |
|  |  |  |  |
| Game of life | Sad old age | Find an alternative | Not clear to many |
|  | Love others | Being kind | Love is easily interpreted to romantic relationships with opposite sex. |
|  | Big business | Find a better replacement | Uncommon for an adolescent in Uganda to own such. |
|  | Lose everything | Lose money | Everything may not make sense to an adolescent. |
|  | Be a loner |  | Not understood |
|  | Common adolescent issues include: betrayed by a friend, ashamed, left out in an activity, bullied at school | | |
|  | Take from others | An alternative should be used. | More like stealing which is already listed. |
|  | Abandoned by family | Neglected | The alternative sounds better. |
|  | Be honored | Respected | Respect is more meaningful to an adolescent than honor |
|  | Wealthy | Rich | Rich is a more common word. |
|  | You stink | Smell bad | Not every adolescent will make sense of stink. |
|  | Love | care | We often don’t discuss love issues with adolescents since it automatically translate to intimacy |
|  | Experience beauty | Look beautiful | Experiencing beauty is unclear in meaning. |
|  | Fame | Become famous | Alternative is action oriented. |
|  |  |  |  |
| Emotional cards | Shocked | alternative | Does not bring out shock |
|  | Calm | Alternative | Not liked |
|  | Disregard/contempt | Alternative or remove | Word isn’t clear and photo does not bring out the meaning. |
|  |  |  |  |
| Strength cards | Being fair | A picture involving sharing of things could be better. | Hugging does not represent being fair. |
|  | Humility | Change picture | Picture does not match the narrative. |
|  | Wisdom | A better picture | Current picture represents talent not wisdom. |
|  | Being grateful | Change picture | Not very appropriate. |
|  | Capacity for love | We can change the world | Not easy to understand |
|  | Curiosity | Desire to know | The word may not be easy to understand by adolescents. |

- Success scenarios also need to change because they are limited to school experiences yet we also have many adolescents who are out of school. Besides, some do not represent typical experiences of Ugandan life.
