# Supplementary material for: Culturally adapting a mindfulness and acceptance-based intervention to support the mental health of adolescents on antiretroviral therapy in Uganda
Source: PLOS Glob Public Health. 2023 Mar 7;3(3):e0001605. doi: 10.1371/journal.pgph.0001605 (PMC10021405; doi:10.1371/journal.pgph.0001605)
Supplement: S9 Data — (DOCX) [file pgph.0001605.s011.docx]

| **Bernal’s category** | **(1) when and how in the implementation process the modification was made.** | **(2) whether the modification was planned.** | **(3) who determined that the modification should be made.** | **(4) what is modified.** | **(5) the reasons for the modification** | **(6) type or nature of context or content-level modifications made.** | **(7) the extent to which the modification is fidelity-consistent.** |
| --- | --- | --- | --- | --- | --- | --- | --- |
| Metaphors and Language | Stakeholders workshop with local mental health experts and practitioners. | Planned modification | A group of local mental health experts and practitioners suggested modifications through consensus | The language of the protocol is slightly advanced and technical for adolescents in Uganda. Replace some words with simpler synonyms, also translate to Luganda the most spoken language. Adolescents should provide words they relate with most | Much as English is the official language in Uganda, it’s not the first language. Besides, some adolescents with HIV are illiterate and their comprehension of English is limited. Also some may not have the cognitive ability to understand some terms. | Surface level modification |  |
|  | Stakeholders’ workshop with local mental health experts and practitioners. |  | A group of experts suggested modifications through consensus | Where the manual suggest use of videos, these should be replaced with narratives by the facilitator or plays!  For example, suggested You-tube videos like “fitting in”, the message can be said out or acted. | Accents used in videos are hard to understand or be comprehended by adolescents. Secondly, gadgets and internet need to show You-tube videos are not available at most public facilities. Besides, adolescents spending less time at clinics, thus, use of videos is not realistic in a local setting. | Surface level modification (metaphors and aids). |  |
|  |  |  | A group of experts suggested modifications through consensus | Change metaphors that involve use of ideas, products, or items which are unknown in Uganda.  **(*i) “the sea weed metaphor***” This should be changed to papyrus in a swamp or a branch on a tree under strong wind. | When metaphors are built around items, products or ideas which are alien in the context, adolescents will not relate with them. Case in point is:  We don’t have seas in Uganda and adolescents may not relate well to what the sea weed is. | Surface modification (metaphors) |  |
|  |  |  |  | **(ii) *“The game of life”***  Change some destinies mentioned in the game to make it closer to reality in Ugandan context. Some terms used are un-relatable for adolescents “scrooge, get fired, be a hermit, embezzle money”. These be replaced with words adolescents understand. Engage adolescents to elicit understanding and where possible elicit synonyms. | Some destinies are stated in terms which are not easy to understand or would be, misinterpreted by adolescents. Furthermore, some are not common use or even familiar to less educated adolescents and some are out of reach for their age. People who have never worked before may not feel the pain of getting fired. Certain experiences like going for a vacation are not common occurrences in low income contexts. | Surface adaptation (language and metaphors). |  |
|  |  |  |  | **(ii)**The “survivor game”, Replace the warrior with a hunter getting confronted by wild animals. the story of a warrior does not relate so well”. | Warrior stories are uncommon around. Few adolescents relate to who a warrior is but many know who a hunter is in a typical African setting. |  |  |
|  | Workshop with adolescents living with HIV | Planned modification | A group of adolescents living with HIV agreed on modification | The game of life metaphor should also include aspects like: keeping close with friends, getting closer to God, being in good terms with family, joining University rather than college, listening to music | Such mentioned items make good meaning and are more relatable. |  |  |
|  | Workshop with adolescents living with HIV | Planned modification | A group of adolescents living with HIV agreed on modification | Further still, the game of life has items which should be replaced or simplified. Items to be replaced include: Get deported, impulsive and graduate high school. Items to simplify include: Being grumpy=short tempered, face ruin=lose everything, graduate high school=complete secondary school, be a hermit= be alone, scrooge=miser, lie compulsively=, embezzle money=steal money, be impulsive=rushing, Persist=don’t give up, love others=like others. | Some words are out of context, for example “deporting”, while others are hard to understand by adolescents watering down the intention of the metaphor. |  |  |
|  | Stakeholders workshop with mental health experts. | Planned modification | A group of experts suggested modifications through consensus | Free hugs in Santori. Be replaced with a request for a high five or “bonga” or simply a more relatable gesture. O | Hugging strangers is culturally unacceptable and religions like Islam to which some adolescents subscribe are against opposite sex hugging. |  |  |
|  | Stakeholders workshop with mental health experts. | Planned modification | A group of experts suggested modifications through consensus | The poem by Bo Burnham under “my advisor sounds like activity.”, be removed and replaced with simple words written on paper. Following prompts: request adolescents to: 1-read without making sound, 2-read out loud and 3-read with a broken voice: They do all this while listening to what their advisor says. Prompts could include voices that sound like famous personalities in Uganda. | Much as the message is important, the poem is not clear, not relatable, names identified under prompts are unfamiliar (George Costanza, Eddy Murphy etc.). The intended objective of having the poem does not come out. |  |  |
|  | Stakeholders workshop with mental health experts. | Planned modification | A group of experts suggested modifications through consensus | Incorporate metaphors that relate to living with HIV as a condition. For example “swallowing a pill”, visiting a clinic. | Since the therapy is being adapted for adolescents living with HIV, bringing experiences closer to what they are undergoing will resonate better. |  |  |
|  | Graduate student’s workshop. | Planned modification | Graduate students (counselling psychology and Clinical psychology) | Include a cartoon pictorial of an adolescent on a life journey with “a value” at the end and then possible challenges that are mostly faced by adolescents living with HIV as possible bumps on the journey and then include DNA as potential choices. This should be displayed in the opening session. The curiosity of DNA can now be addressed with sessions. | Adolescents with HIV are a category that is already undergoing enough strain in life, thus, getting their attention is sometimes hard. Thus, depicting how the entire story is going to look like in a pictorial can attract their attention and also increase interest in therapy. Besides, adolescents are concrete thinkers: taking information at face value with less cognitive effort, thus, simplicity in representing the message is the only way to make it practical. |  |  |
|  | Graduate student’s workshop. | Planned modification | Graduate students (counselling psychology and Clinical psychology) | The protocol addresses participants as “students”. This should be changed to adolescents. | Not all adolescents are in school. Some are working and others married. |  |  |
|  | Graduate students workshop. | Planned modification | Graduate students (counselling psychology and Clinical psychology). | Use the word “awareness” in the place of “noticer” most times. Could be awareness of how we feel right. | Awareness is closer in meaning to common day to day words adolescents in Uganda use than noticer. Noticer makes the DNA complete but can be used at introduction and then “awareness” takes over for purposes of relatability. |  |  |
|  | Graduate students workshop. | Planned modification | Graduate students (counselling psychology and Clinical psychology). | Replace the following high level words with relatable terms: (I) Unhook be replaced with detaching or separating from experiences. (II) Sensations: how you feel. | These are important concepts in the therapy but risk not being understood by adolescents who are not entirely English speakers. |  |  |
|  | Graduate students workshop. | Planned modification | Graduate students (counselling psychology and Clinical psychology). | All you-tube videos be removed from the metaphors and rather be replaced with images that portray the same message. For example, “free hugs in Santorini” and “Baby responding to dad evil laugh” be replaced with either a crying face, someone helping a vulnerable person or any other emotional provoking image. | Internet is expensive and unsustainable in clinics where sessions will be held, thus, therapy relying on such demands is not feasible. Besides, videos along prolong duration of therapy affecting concentration and interest. |  |  |
| Content | Stakeholders workshop with mental health experts. | Planned modification | A group of mental health experts suggested modifications through consensus. | Noticing and expression of feelings need to be buffered with pictures or drawing relating to the message. With the current arrangement, adolescents many struggle to relate. | Most adolescents may not know how to express or describe their feelings. Without proper expressions and aids to guide appreciation of noticing, the message may easily be lost. We are in a culture where noticing is not well nurtured. |  |  |
|  | Stakeholders workshop with mental health experts. | Planned modification | A group of mental health experts suggested modifications through consensus. | Besides values and strength cards, **emotional** **cards** with faces expressing varying emotions should be designed and added to the package. | This will help adolescents put name to their emotions. This offers a starting point to the facilitator to track and develop and name the emotion expressed. Much as expression of emotions is presumed to be universal, interpretation may differ. Also Ugandans are not good at naming emotions, thus, a guide in form of cards will be useful. |  |  |
|  | Stakeholders workshop with mental health experts. | Planned modification | A group of mental health experts suggested modifications through consensus. | More visuals, cartoons or plays should replace written content where necessary. | The language of the protocol is unfamiliar to most adolescents and if not well managed might be misrepresented as an informative training session. These adolescents are undergoing many life struggles, thus, sitting in an informative class is among the last things to be happy about. |  |  |
|  | Graduate students workshop. | Planned modification | Graduate students (counselling psychology and Clinical psychology). | The “noticer” should be dramatized, acted out in plays and brought out in real life or represented in cartoon character. | This is the hardest component of the protocol to understand. The language and process is unfamiliar. We are in a culture where expression of feelings is not common. Adolescents with not make a lot of sense out of it unless its presented in the simplest way possible. |  |  |
|  | Stakeholders workshop with mental health experts. | Planned modification | A group of mental health experts suggested modifications through consensus. | Homework tasks be tied to their lived conditions. For example, task them to notice what their advisor says whenever it’s time to taking medicines. | Relating to their condition brings the therapy closer to them as a lived experience rather than leaving it in generics. |  |  |
|  | Graduate students workshop. | Planned modification | Graduate students (counselling psychology and Clinical psychology). | Add a mindfulness exercise ahead of introducing the Noticer. This should be a step by step process to create familiarity and awareness. It should entirely be acted out. Eating bananas mindfully or filling balloons to watch breathe might work. | The whole process is novice and need better grounding to be appreciated. |  |  |
|  | Graduate students workshop. | Planned modification | Graduate students (counselling psychology and Clinical Psychology) | When discussing discoverer moves, make a mention of some risky behaviors adolescents with HIV are likely to try out: missing medicines, missing clinic appointments, beating up someone trying stigmatize you or walking away from a support group. | Using lived experiences brings the therapy closer to users. Adolescents living with HIV will relate more to such examples. |  |  |
| Methods of Delivery and context. | Stakeholders workshop with mental health experts. | Planned modification | A group of mental health experts suggested modifications through consensus. | Opening sessions should be more about building rapport, trust and closeness. Not immediately into intervention content. | Uganda is more of a high context culture. Strong interpersonal bonds at the start will influence attention and retention in therapy. |  |  |
|  | Stakeholders workshop with mental health experts. | Planned modification | A group of experts determined modification through consensus | All videos throughout sessions should be replaced with skits led by facilitators to replicate the message in the video. | Facilities where sessions are targeted to be held may not have the luxury of gadget to display videos, secondary constant power blackout in Uganda will affect sessions and besides, the English used in videos might be hard for some adolescents to understand. Acting videos out will also make sessions more participatory. |  |  |
|  | Workshop with adolescents living with HIV | Planned modification | A group of adolescents living with HIV agreed on modification | Story telling should be used more often. Where possible, let content be presented as stories. | Story telling is less tasking cognitively, makes it easy to relate to an individual’s personal experience and sparks more participation. |  |  |
|  | Stakeholders workshop with mental health experts. | Planned modification | A group of mental health experts suggested modifications through consensus. | Sessions be made to last one and half hour not two hours as planned. | Concentrating for 2 hours might be long for adolescents who already have underlying conditions. Besides, when sessions are very demanding attrition will increase. |  |  |
|  | Stakeholders workshop with mental health experts. | Planned modification | A group of mental health experts suggested modifications through consensus. | Group size should be 6-12 and the age factor be considered in group formation. Adolescents 14-17years should be in a separate group and the 18-19years also in a separate group. | Any group beyond 12 adolescents is ineffective. The best practice around is to have 6-12 for therapy related work.  Secondly, adolescents 14-17 are at a different developmental stage and still considered minor is Uganda. Putting them in the same group with adolescents (18-19years) who are considered as adults in Uganda compromises their participation and independence to express. In Uganda older people have more authority than young ones and that should be reflected even in participation. |  |  |
|  | Stakeholders workshop with mental health experts. | Planned modification | A group of mental health experts suggested modifications through consensus. | Sessions be compressed a bit to fit in a short span. Six weeks are many for adolescents who have to commute from home to clinic. 3 to 4 meetings are better. | It becomes costly for adolescents to move to clinics to attend therapy sessions. For the study where transport refund is made, this becomes possible, however for real life use, not many will afford moving to clinics to attend all sessions. Considering school times and logistical issues is important in a low resource setting. Also clinics where sessions will be held have a challenge of space, thus, running many sessions is not feasible. Will create competition on space. |  |  |
|  | Stakeholders workshop with mental health experts. | Planned modification | A group of mental health experts suggested modifications through consensus. | The last session be focused on relatability and moving out into real life. It should entirely focus on making recaps of what adolescents have learnt, if they are practicing and how they are utilizing the learnt skill. | In Uganda most learning is left where it happened. Without proper relating and bringing it to life, chances of relapse are high and most likely the skill will never be put to use. |  |  |
|  | Stakeholders workshop with mental health experts. | Planned modification | A group of mental health experts suggested modifications through consensus. | Homework assignments should be closely related to adolescents’ condition so that it’s given attention. Since the intervention is targeting adolescents living with HIV, any assignment relating to the condition is given priority.’ | This will ensure that the assignment is done as self-help not merely to appease the facilitator. Once they gain understanding on how its helping, they will do it with ease. Some exercise like pausing to notice, taking a breath are not commonly used here, thus, nurturing them needs a connection to something very important to the participant. |  |  |
|  | Stakeholders workshop with mental health experts. | Planned modification | A group of mental health experts suggested modifications through consensus. | Utilize clinic visit days to schedule sessions. Drama clubs at clinics can also be used to act out some concepts of the treatment. | The idea of utilizing adolescent clinics will make the entire process practical. Most adolescents are attending school, thus, they need a flexible schedule. |  |  |
|  | Graduate students workshop. | Planned modification | Graduate students: Clinical Psychology and Counselling Psychology | Open and end all sessions with prayer. Include prayer among the opening remarks before recaps on previous session. | Ugandans are very prayerful people and adolescents living with HIV find a lot of solace in being closer to God. |  |  |
|  | Graduate students workshop. | Planned modification | Graduate students: Clinical Psychology and Counselling Psychology | Deliver sessions as a road map. Create a journey that adolescents can relate with from the start to the end. Orient adolescents from the start about this journey and the different concepts are stop overs on the journey. | With the current arrangement of the protocol, adolescents may not be able to connect parts. Even though recaps help to connect, sessions still look like detached components. |  |  |
| Values and practice. | Stakeholders workshop with mental health experts. | Planned modification | A group of experts suggested modifications through consensus. | All cards used in sessions “values and strength” Should carry relatable messages and images. Both words and pictures | Some of the messages represent an unfamiliar life to a Ugandan adolescent. If these are to be used, they need to communicate reality of a Ugandan setting. |  |  |
|  |  |  | A group of experts suggested modifications through consensus. | Adolescent cherished values be used. Most appropriately those that appeal to adolescents with HIV. | Some listed values will not be selected because they are alien to the context. Engage adolescents to agree on what makes meaning to them. |  |  |
|  | Workshop with adolescents living with HIV |  | A group of adolescents living with HIV agreed on modification | Accepting self, being closer to God and Gratitude were the main values mentioned by adolescents. | Most of the adolescent living with HIV consider these to be important when coping with their condition. |  |  |
|  | Workshop with adolescents living with HIV | Planned modification | A group of adolescents living with HIV agreed on modification | Make narratives or give stories of real life situations and from the stories show what the particular value was in that situation. | Values were not identified easily by the adolescents, they need help. Most of them confused values with hobbies or likes. |  |  |
| People | Stakeholders workshop with mental health experts. | Planned modification | A group of experts suggested modifications through consensus. | Counselors working in HIV clinics should be the targeted people to deliver the therapy sessions. | For purposes of scalability and continuity, delivery of DNA-v sessions ought to be included in the daily routines of providers at the clinic. Adolescents living with HIV have closer relationships with their care providers than outsiders walking into the clinic and besides, resources to support an outside practitioner are never available. |  |  |
| Negotiating differences in Knowledge | Stakeholders workshop with mental health experts. | Planned modification | A group of experts suggested modifications through consensus. | Facilitator must have live experiences of mindfulness to share with adolescents. Where they have used it and how it worked for them. | Mindfulness or noticing are not common practices in Uganda, thus, facilitators need to begin with getting of a clear understanding on the concepts and relate to their lives. Adolescents relate more with shared lived experiences rather than logical appeals. |  |  |
|  |  |  |  |  |  |  |  |

REVIEW STAGE

Two review were made, first by a selected group of local mental health experts and providers (5) and a selected group (5) adolescents living with HIV. All participants were selected from the groups that participated during generation.

| Suggested further modification | Rationale | Who determined the modification |  |
| --- | --- | --- | --- |
| Add a preamble to the manual and provide more details about DNA-v to guide the user early enough. | Ugandans have a short attention span and easily lose the spark once they start to struggle with concepts. Secondly, some people might be interfacing most concept for the very first time, thus, no need of keeping them in suspense. | Local mental health experts (5): All the 5 agreed to this. |  |
| Move reference materials to the end of the protocol. They suggest an unnecessary baggage to users who are most likely reluctant to follow up. | Ugandans don’t have a reading culture. They are most interested in how it works and how best do we get it working other than knowing the theory surrounding the intervention. Bringing it early subjects users to reading biases. | 3 of the 5 local mental health experts agreed to this suggestion. |  |
| Change cover page to look familiar to a local context and intentionally utilize pictures that help one make sense of the manual before detailed reading. | Most people make decisions to read manuals basing on the appearance of the cover page. There is also strength in first impression, thus, the page should be able to say it all. | 5 out 5 all agreed to this suggestion. |  |
| Sequencing of sessions; offering one session in a day, spread across six weeks looks ideal but will not work for adolescents living with HIV. Reduce sessions to three and try to cover two topics per meeting and each meeting should not go beyond two hours. Role plays and metaphors should dominate the session. | Some adolescents come from places far away from the clinic which ends up being expensive in terms of transport while others want to minimize the number of times they come to the clinics as a way of managing stigma. If delivery is made from a school setting the original scheduling works, however in a real life clinic setting it may not work. | All 5 reviewers agreed |  |
| Maintaining the word “Noticer” rather than replacing it with awareness as it had earlier been suggested. | Reviewers observed that much as the original idea sounded proper, since “N” is a strong pillar in DNA-v cannot risk replacing Noticer with awareness in the protocol. Noticer should be retain and then awareness indicated in brackets for every stage the word Noticer come into use. | 4 out 5 had consensus about this while 1 still felt that NOTICER is a harder term to remain the manual. |  |
| Language of the entire manual be revised and simplified without translating it to Luganda. All difficult words be replaced with simple one that are at a basic level of language proficiency. | The manual is to be used by counselors to offer support to adolescents, and since most are educated and understand English, it does not necessary require translation into Luganda. Besides, the ethnically diverse nature of Uganda as a country does not permit translation to just one language. |  |  |
| Success scenarios be remodified: photos and stories depicting success be changed to reflect practices in Uganda and common things adolescents are more familiar with. Suggestion to engage adolescents to come up with these scenarios were made in the first review. During the second review, adolescents came up with the stories and also gave input on appropriateness of the photos/pictures used. | For the success stories to make sense, they have to be familiar to lived experiences of adolescents. They should be able to imagine themselves in such situations. | 5 out 5 reviewers agreed to the suggestion to have success scenarios reviewed. |  |
| Opening and ending sessions with prayer was debated by reviewers and observed that not everyone in the audience is presumed to be religious, thus, its suggested that adolescents be consulted on how they would want the session to be opened/started. | Prayer is central to many people in Uganda but to avoid inconvenience to those who are not prayerful, making an open request to adolescents on how they want the session to be open up is more inclusive. | 3 out of 5 reviewers agreed to this suggestion although 2 had insisted on making prayer mandatory while opening and closing sessions. |  |
| Re-emphasizing group confidentiality across all sessions. At the start and end of every session, participants be reminded about the need to maintain group confidentiality. Participants also be notified about possibility of breach since some members might act centrally. | These are young people living with a chronic condition, thus, their protection is of paramount importance. Besides, being group therapy, possibility of breach is high. This notification will help them decide on how much of private information they can give. | All 5 reviewers agreed to this. |  |
