# Supplementary material for: Culturally adapting a mindfulness and acceptance-based intervention to support the mental health of adolescents on antiretroviral therapy in Uganda
Source: PLOS Glob Public Health. 2023 Mar 7;3(3):e0001605. doi: 10.1371/journal.pgph.0001605 (PMC10021405; doi:10.1371/journal.pgph.0001605)
Supplement: Attachment — Submitted filename: Response to reviewers.docx [file pgph.0001605.s012.docx]

The Chief Editor

PLOS Global Public Health

December 5^th^, 2022

**Re-submission of our Manuscript “Culturally adapting a mindfulness and acceptance-based intervention to support adherence to antiretroviral therapy among adolescents with HIV in Uganda” PGPH-D-22-01531**

Dear Editor-in-Chief,

I thank you for the opportunity given to us to revise and resubmit our manuscript. I also thank the reviewers for the constructive feedback they shared with us which has helped to improve the quality of the manuscript. We have given careful attention to every comment and I believe the transcript is in a strong position to meet PLOS Global Public Health’s publication criteria. Responses to reviewers’ comments are presented in the table below:

| REVIEWER 1 | | |
| --- | --- | --- |
| Abstract | **Line 15**: It is not clear who are these stakeholders – please add details (e.g., local mental health experts, spiritual and adolescents with HIV) and how many stakeholders were engaged (n=30).  **Line 21**: how many adolescents were recruited for qualitative interviews?  **Line 24**: suggest to say “the adapted intervention was perceived as acceptable among…” | Stakeholder details and the number have been added to Lines 15-16  We now indicate in Line 22 that nine adolescents were recruited.  Addressed in lines 26 – 27 |
| Introduction | **Lines 96-98**: the authors say that research supports the effectiveness of DNA-v among adolescents with chronic conditions – does this include HIV? If not, the introduction should mention this and the need to adapt the DNA-v intervention to adolescents with HIV (in addition to cultural and contextual adaptations).  **Line 100**: please outline some specific difficulties psychotherapies encounter in non-Western settings (e.g., language, culture, etc.)    **Line 103**: the authors note that limited studies have adapted evidence-based psychosocial interventions for AWH – where and how have these studies been conducted and how were the interventions adapted? Please clarify.  **Lines 111-112**: Where will the larger study take place (e.g., in mental health clinics in and surrounding Kampala)? | We now indicate in Line 100 that the DNA-v has never been used with AWH before.  Difficulties which include differences in language, values, the social construction of disease and challenges with supervision have been added in Lines 106-108.  We now provide an example of a study from South Africa that adapted an EBI for AWH. The intervention was adapted based on stakeholder engagement and interviews (please see Lines 111-115)  The larger study will take place in public health centers in Kampala. Line 129-130. |
| Methods | **Lines 114-115**: Add information on how participant consent was obtained for both studies. Study 1 mentions written consent, but study 2 does not provide this information.  **Lines 148-149**: DNA-v was selected as it may improve young people’s mental health. But where and how was this information demonstrated? Add details from the sourced studies – for instance, in what setting and context DNA-v studied? How was young people’s mental health assessed (e.g., self-reported, validated scales, etc)? | Information on consenting participants in study 2 is now provided in Lines 315-317  Details of where the DNA-v has been used before are provided and the rationale for use in Uganda is clarified and expanded in Lines 167-168. |
| Study 1 | **Lines 167-168**: why were only six components of the EVM used? What is the reason for not including the other 2 components as per the EVM?  **Lines 184-185**: provide median age and standard deviation rather than average age. The authors state that stakeholders were majority female (n=18) – it is questionable whether 60% (18/30) should be labelled as a “majority”.  **Lines 201-212:** The authors should provide more detail on how many workshops were conducted and with whom. It is not clear which stakeholders are considered “local mental health providers and experts” – does this include all stakeholders except AWH? Does it include spiritual leaders?  Moreover, how long was the workshop with AWH? Was the workshop also recorded, transcribed and translated? | EVM components have often been criticized for overlapping, thus, we adopted the 6 components that had been used in a related study in Uganda. This is mentioned in Lines 195-196.  The median age and standard deviation have been provided for both samples and the language used have been revised. Please see Lines 214 - 215.  This has been clarified. Two workshops were conducted at generation (1=experts and 1=AWH) whereas other meetings were for review. Please see Lines 249-250.  Spiritual leaders were considered under local experts and practitioners. Please see Line 238  The AWH workshop lasted for 3 hours (see Lines 248-249). The procedure for dealing with information generated in workshops is provided under the analysis section of Study 1 (Lines 278-283) and the integration section (Lines 253-261) |
| Study 2 | **Pages 20-21** (procedures): it is not clear whether all 9 AWH who were interviewed attended all six DNA-v sessions. Moreover, how long were the in-depth interviews? Were they conducted one-on-one with a researcher? Can the authors provide examples of questions from their interview guide (if interviews were semi-structured)?    **Page 21 (analysis):** The description of the analysis is brief. The authors previously mentioned that questions were guided by a theoretical framework – how did this influence the analysis? In stage 3, please clarify what is meant by a “set of themes” – does this relate to a coding scheme (or sets of codes)? Were no new codes added to the coding scheme during analysis of the six remaining interviews?  **Page 23 (results):** the final sentence of the results describes that the authors synthesized findings and made a final round of refinements, but no other detail is provided. A table of summarized findings and final refinements should be included. | All AWH attended the six sessions (please see Line 321).  In-depth interviews lasted between 25-30 minutes (see Line 322).  Interviews were one-on-one and conducted with a member of the research team. Line 326 – 327.  Sample questions are now provided in Lines 305-308.  The analysis has been expanded to provide more detail. Line 332-347.  “Table in S2 table” (supporting information) now shows the additional refinements after the testing phase. Please see Line 533 |
| Discussion | The discussion is limited to the adaptation of the DNA-v to AWH (study 1). However, there is little mention of the “testing” of the DNA-v with 9 AWH participants and results from in-depth interviews.  The authors should discuss limitations to where and how the DNA-v intervention is delivered. For instance, as ART adherence is sub-optimal in adolescents, those who seek treatment regularly at a health centre may already have better adherence and HIV knowledge than those who do not visit health centre regularly.  Authors should discuss broader structural factors that may impact AWH's mental health and well-being. For instance, self-acceptance and self-stigmatization may be improved if HIV knowledge, acceptance and HIV stigma was addressed amongst young people not living with HIV. | The discussion has been expanded to cover ground for phase2 of the study (Line 593-612), we were however mindful of the length of the paper.  Limitations related to where delivery of sessions happen have been added and clarified. Line 642-647.  The aspect of broader structural factors affecting mental health of AWH have been recognized and discussed. Line 603-613 |
|  | | |
| REVIEWER 2 | | |
| Overall | Sub-study 2 is less persuasive than Sub-study 1 as an independent element. The overall argument and presentation could be strengthened by integrating the sub-studies with a usual presentation format e.g., methods, results, discussion.  As AWH were also interviewed for sub-study 1 and these discussions had important implications for study location e.g., that schools are not good sites for interventions due to stigma, findings from across adaptation discussions and AWH qualitative interviews perhaps could be coded and integrated/reported as part of AWH qualitative findings. This could provide more substantiated support for the results sub-section reporting on actions taken to improve adaptation. Practically this would involve integrating the methods into one section, and presenting the results in a more comprehensive way with qualitative findings first, then adaptation actions second. In its current form, results from AWH coded interviews could be expanded. | We appreciate this suggestion and have integrated the sub-studies and presented it in the usual format. Please see Lines 189-455 and 332-572  Findings that influenced adaptation were generated through consensus and majority vote. The approach is supported by several adaptation frameworks and studies (see Lines 278 - 284). Thus, feedback from the adaptation phase was not analyzed in the usual qualitative format.  Qualitative findings from Phase 2 have been expanded as suggested in Lines 455-526 |
| Additional | **P.4, line 54**. Prior to the statement "more than half of the new infections"... no information was given on the number of new infections globally, in SSA, and in Uganda. It would also make sense to state the prevalence in SSA, and in Uganda overall, and among young people. From here on, the introduction does an excellent job presenting the necessary context. | Information on new infections and prevalence in Uganda is now added to the background in Lines 56-61. |
|  | **P.10, line 188**. It would be helpful to know how the participants were selected within stakeholder categories e.g., purposive selection, convenience, targeting gender balance or balance of other characteristics or experiences? | Clarification on how participants were selected in Phase 1 of the study has been provided in Lines 222-225 |
|  | **P.10, line 215**. What was the step between recording of discussions and synthesis e.g., how were themes collected and collated in a uniform way from recordings and transcriptions e.g., coding framework, like the second sub-study? | Intra- and inter-group consensus was obtained and calibrated with literature and practice before guiding modifications, as detailed in Line 258-264. |
|  | **P. 16.** Would translation have to occur into all 41 Ugandan dialects? Given this work is taking place in Kampala, it might have been beneficial to translate/back-translate the manual into the most commonly spoken language within Kampala (e.g., Luganda and/or Swahili) in order to ensure that the semantics were captured. I think the approach taken is nonetheless acceptable. p.17. Without a translated version, adding local words and slang into the manual seemed like an appropriate way to facilitate semantic understanding. | Due to logistical constraints, we were not able to translate the manual to the most spoken languages. We however appreciate the reviewer’s observation that adding local words and slang is an appropriate way to facilitate semantic understanding. |
|  | **P.20.** Participants. Same question as above on participant selection - please clarify the approach. | We now clarify how participants were selected for Phase 2 in Lines 309-311. |
|  | **P.20**. Procedure. How were IDIs conducted? Was a topic guide used? If so, please include. If not, please describe the approach. How long did interviews last (time range)? | IDIs were guided by an interview schedule developed from the constructs of the theoretical framework of acceptability (Lines 326-327).  Interviews lasted between 25-30 minutes (Line 322). |
|  | **P.21**. I believe this is the first time reading about "acceptability-TFA" and not sure what it is (a search does not bring up a prior use). If this is the major theoretical framework used for analysis, it should be introduced and described in the introduction or methods. | The TFA is now well covered in the introduction and expanded in the methods (please see Lines 123-126). |
| Results | **P.21**. It would be helpful to introduce this section with a 1-2 lines of overview of the major themes identified. At the moment, the findings presented seem somewhat thin, and could be further extrapolated. Currently, only positive elements are reported. Did participants offer any constructive feedback either relating to their own experience, or perhaps something related to how they perceive broader acceptance of the intervention? Were there outliers, or contrasting views?  Did participants report anything relating to their perception that the intervention could help to encourage adherence to ART? | Results are presented under *a* *priori* themes of the Theoretical Framework of Acceptability (Lines 460)  The Qualitative results have been expanded in Lines 462-526.  Contrasting views regarding ease of understanding the DNA-v are represented. Line 516-519.  Yes, participants did report that the intervention could help to encourage adherence to ART. This information is now provided in Lines 463-468. |
| Discussion | At this stage, it might be helpful here to give some examples of successfully adapted interventions that were also evaluated and demonstrated evidence for effectiveness | Examples have been included in Lines 111-114. |
|  | **P.24,** top. "These findings..." Is this line referring to the present findings (e.g., author's work) or findings from the study cited in the line before? What is meant by "non-dominant cultures?" Does this mean the majority ethnicity? | The statement on which findings are being referred to has been clarified in Lines 559-560.  The statement on “non-dominant cultures” has been revised in Line 564-565. |
|  | "Our findings suggest that low-resource settings have structural barriers..." This is absolutely true, but I did not take that message away within the context of the present work from either sets of results presented. If participants discussed such barriers, consider adding these findings to the results section(s) to facilitate this part of the discussion. | Structural barriers are mentioned in “Table in S1 table” (supporting materials) which summarizes the results from Phase 1. More specifically, the headings “Methods of delivery”, and “Social context” are themes of the ecological validity model. |
|  | **P. 24**, bottom. The "most interesting findings stated here" are not evident in results. This may reinforce the feedback that the results section for study 2 should be expanded and perhaps re-organized. | Results from Phase 2 of the study have been expanded in Lines 462-529. |
|  | **P.25,** first para. Correct syntax in the last line. Also, rephrase slightly for clarity: "...acceptability to be a multifaceted construct that should be constructed beyond its constructs..." | This has been clarified in Lines 615-620. |
|  | **P.26**. "...our study found schools to be stigmatizing environments..." This is an important finding but one that is not presently well substantiated in the presentation of results from adaptation discussions as they are not currently coded and analyzed. It would be helpful to get a sense of the points of agreement within and across stakeholder groups on adaptation actions, and any outlier views, from these discussions. | These results are presented in “Table in S2 table” which summarized the adaptation results (Line 532).  Feedback from Phase 1 of the study was based on consensus and majority vote as explained under the analysis stage for Phase 1 (Lines 278-284) and integration stage (lines 258-262). |
|  | **P.26**, bottom. This is an important point on involvement of parents/guardians. It would be helpful in methods to clarify local requirements for consent/assent. For participants who assented, was consent of parent/guardian required? If not, how did assent process work for minors (add to page 10, line 185) | Local requirements for consent/ assent have been highlighted and a rationale for obtaining parental consent provided in Lines 228-231. |
|  | Ref. 37. Is there a URL? Not sure there is enough information provided to track this ref down. | The URL for the reference (41) has been added in Lines 800-801. |

Sincerely,

Khamisi Musanje

Principle Investigator
