# Supplementary material for: Culturally adapting a mindfulness and acceptance-based intervention to support the mental health of adolescents on antiretroviral therapy in Uganda
Source: PLOS Glob Public Health. 2023 Mar 7;3(3):e0001605. doi: 10.1371/journal.pgph.0001605 (PMC10021405; doi:10.1371/journal.pgph.0001605)
Supplement: Attachment — Submitted filename: Response to review.docx [file pgph.0001605.s013.docx]

The Chief Editor

PLOS Global Public Health

January 16^th^, 2023

**Re-submission of our Manuscript “Culturally adapting a mindfulness and acceptance-based intervention to support the mental health of adolescents on antiretroviral therapy in Uganda” PGPH-D-22-01531**

Dear Editor-in-Chief,

I thank you for the opportunity given to us to revise for the second time and resubmit our manuscript. I also thank the reviewers for the constructive feedback they shared with us which has helped to improve the quality of the manuscript. We have given careful attention to every comment and I believe the transcript is in a strong position to meet PLOS Global Public Health’s publication criteria. Responses to reviewers’ comments are presented in the table below:

| **Reviewer 1** | | |
| --- | --- | --- |
| Overall | Lines 240-246 (Analysis of Phase 1) seems to lack information – did the authors code and analyze transcripts of both group discussions? | In Lines 244-247, we indicate that the data from phase 1 was not qualitatively analyzed but rather used majority consensus to agree on changes. The most recurrent suggestions guided the adaptation. A reference to this approach is also cited in Line 246. |
|  | Lines 292-206 (Analysis of Phase 2): Although the authors state that “data were analyzed using abductive analysis”, the generation of themes seems constrained to a deductive approach. Evidence of an inductive process is lacking. | Additional information related to the inductive process has been added (please see Lines 311 - 325). |
|  | Lines 408-409: The authors report that this phase of the study has been reported in accordance with the COREQ checklist. The COREQ checklist provides guidance for conducting qualitative research. Please complete the checklist in its entirety and include as an attachment to the manuscript. | The COREQ checklist has been completed and attached. |
|  | Lines 412-279 (Results of phase 2): This section remains unclear. The authors have added a table presenting themes and sub-themes; however, the written section is not organized by themes. It is recommended to remove Table 2 and present findings similarly to results from phase 1 (i.e., Theme name: description + verbatim quotes from participants) | Table 2: has been removed and results are presented under the themes of the TFA from Line 430 onward. |
|  | | |
| **Reviewer 2** | | |
| Overall | There remains a disconnect between the title, which refers directly to adherence, and the methods/results/conclusion, which are not at all focused on adherence. The title could be revised to remove reference to adherence while highlighting the original purpose of the intervention (e.g., for adolescent mental health). | The title has been adjusted to represent the original purpose of the intervention |
| Additional | line 124 Capitalize Theoretical Framework of Acceptability? | This has been amended in Line 99. |
|  | line 189. Suggest one methods section with an additional level of sub-heading for phase 1 and phase 2 (e.g., Methods -> Phase 1 ->Cultural adaptation/Participants etc.) | This has been addressed in Line 159. |
|  | line 210-3. Indicate in brackets the number in each category. | This has been added in Lines 181-185. |
|  | line 279. For possible discussion/consideration - discuss possible power dynamics within groups in limitations and how this was controlled/mitigated | Possible power dynamics within groups were considered and have been included in Lines 612-616. |
|  | line 308. Attach a copy of the topic guide. | The interview schedule is now attached. |
|  | line 327. Rephrase for grammar/clarity. | This has been addressed in Lines 298-300 |
|  | line 380. Consider deleting "ghetto" which has specific connotations - is this term typically used in the Ugandan context or is "slum" the best word to describe such deprived urban areas in Uganda? | The term “slum” is more often used to refer to such deprived urban areas. “Ghetto” has been removed from Line 354. |
|  | line 388. Not sure why deleted - I thought this was informative. | This information was deleted because it is part of the final refinements made to the manual which is presented in Table 2. Please see Line 509. |
|  | line 473 (and other places). Usually italics or quotation marks are used, not both. Please check for best practice with editor. | The journal provides no definitive guide on the use of italics or quotation marks in qualitative work. However, a review of its most recently published qualitative papers shows that both italics and quotation marks were used. Please see <https://doi.org/10.1371/>. We have thus retained the use of both italics and quotation marks. |
|  | line 594. Adherence to medication, specifically, or adherence to medical advice in general? Specify. | Adherence to medical advice. This has been addressed in Line 568. |
|  | line 620 The discussion could be improved by including an assessment of quality e.g. validity/transferability of the qualitative findings | This statement has been included in Lines 629-633. |

Sincerely,

Khamisi Musanje
